# Microchemical Analyses of Otoliths Reveal Habitat Differentiation Between a Sympatric Species Pair in the Coastal Waters of China

**DOI:** 10.1002/ece3.73362

**Published:** 2026-04-01

**Authors:** Rongrong Zhang, Kay Lucek, Lisheng Wu, Shufang Liu, Wenwen Zhang, Hongjun Guo, Shaoxiong Ding

**Affiliations:** ^1^ State Key Laboratory of Marine Environmental Science, College of Ocean and Earth Sciences Xiamen University Xiamen China; ^2^ Biodiversity Genomics Laboratory, Institute of Biology University of Neuchâtel Neuchâtel Switzerland; ^3^ Yellow Sea Fisheries Research Institute Chinese Academy of Fishery Sciences Qingdao China

**Keywords:** *Bostrychus*, LA‐ICP‐MS, microhabitat, otolith microchemistry

## Abstract

While secondary contact between distant lineages or sister species is a common evolutionary outcome, the factors that promote their coexistence are often unknown, especially for marine species. Focusing on two sister fish species from the East China Sea, *Bostrychus donghaiensis* and *B. sinensis* , it was assessed whether they differ in microhabitat use and life history using otoliths. Morphological data were used to test for phenotypic differences in otolith morphology between the focal species and employed laser ablation inductively coupled plasma mass spectrometry to measure microchemical changes throughout their life history. The study revealed that otolith shapes not only differ between the two species, but also between *B. sinensis* individuals from within and outside the contact zone. This was further supported by the microchemical analysis, indicating that both *B. donghaiensis* and *B. sinensis* from outside the contact zone primarily inhabit high salinity environments throughout their entire life history without migration. In contrast, *B. sinensis* from the contact zone migrates to estuaries of lower salinity during the breeding season. Overall, these findings highlight the importance of habitat changes in limiting niche competition, contributing to the understanding of life history processes of species during secondary contact.

## Graphical abstract

This study applied otolith morphology and microchemistry to analyze two sister fish species along the China coast. It revealed that *B. sinensis* from the contact zone migrates to low‐salinity estuaries to breed, whereas other groups reside permanently in high‐salinity waters, indicating ecological divergence. These differences in habitat use likely diminish niche competition and may facilitate speciation during secondary contact.

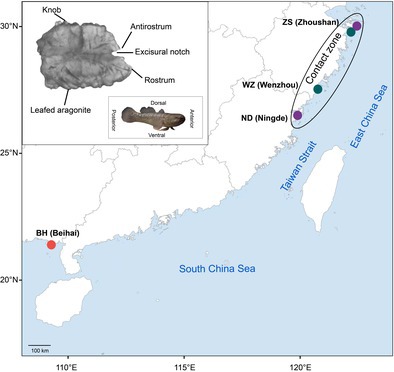

## 1 Introduction

Fish migratory behavior throughout their life history represents an adaptive survival strategy that is influenced by environmental heterogeneity, competitive pressures, and early developmental conditions (Gross et al. 1988; Tamario et al. 2019). Species with divergent evolutionary histories, as well as distinct populations within a species, can employ different migratory strategies (Dodson et al. 2013; Brodersen et al. 2014; Bloom et al. 2018). Spatially co‐existing species may differ in their ecological niches or life histories to limit competition and hybridization (Nosil et al. 2005). Thus, investigating the migratory behavior throughout the life history of sympatric sister fish species offers valuable insights into the ecological factors driving sympatric speciation in marine environments.

**FIGURE 1:**
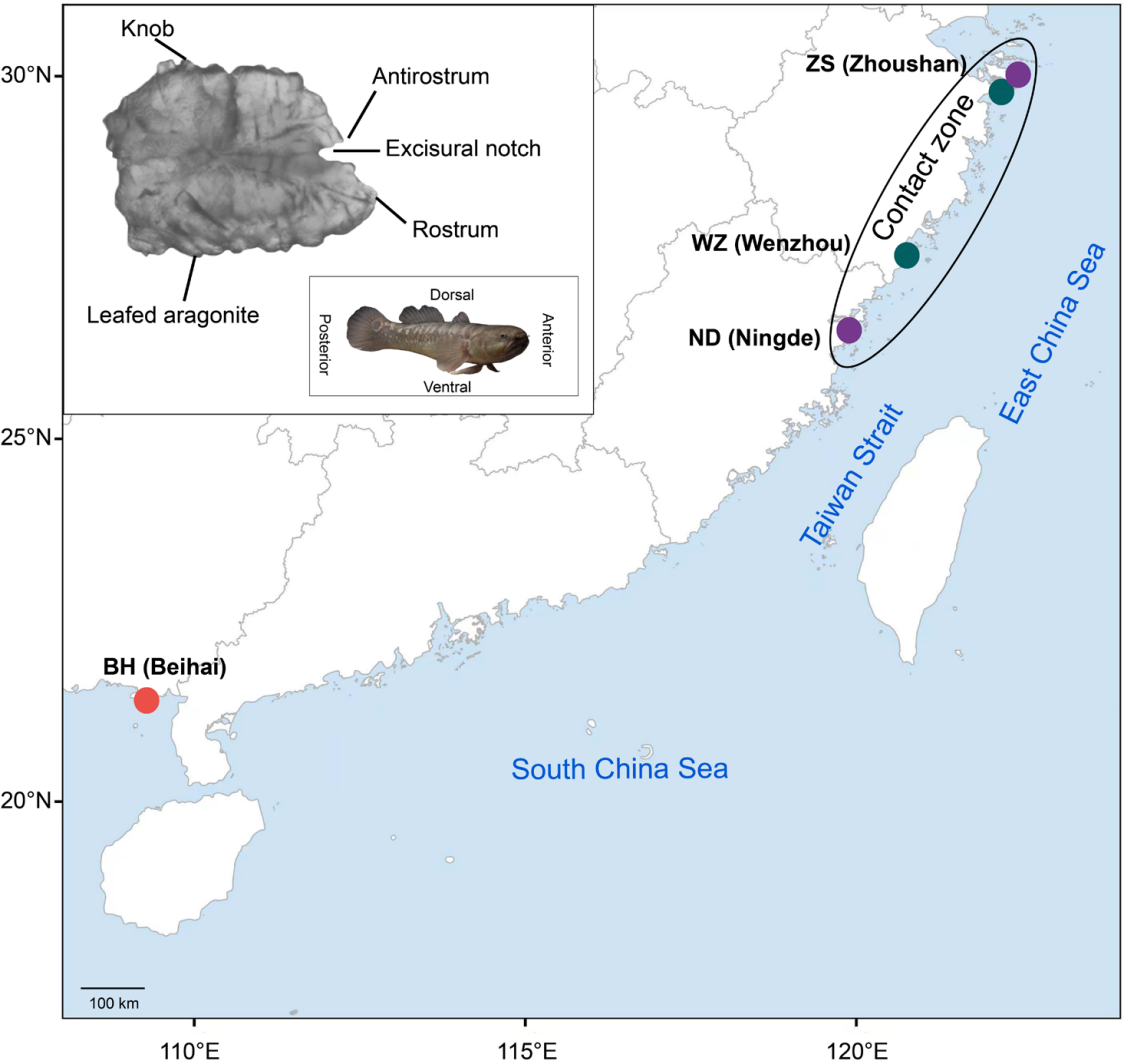
Map of sampling locations for *Bostrychus* along the coastal waters of China. Sampling locations are as follows (from North to South): Zhoushan (ZS), Wenzhou (WZ), Ningde (ND), and Beihai (BH). Colors depict different species and groups: Purple—*B. donghaiensis*; green— *B. sinensis* from within the contact zone (Csin), red— *B. sinensis* from outside the contact zone (OUTCsin). The inset depicts the morphological structure of a sagittal otolith of *B. sinensis* . Morphological traits were defined following Messieh (1972) and Gaemers (1983): Anterior, posterior, dorsal, and ventral correspond to the fish body axes; rostrum: A lamellar projection located at the anterior end of the otolith, extending along the anterior–posterior axis; antirostrum: A lamellar projection located at the posterior end of the otolith, extending along the anterior–posterior axis, opposite to the rostrum; excisural notch: Notch located between the rostrum and antirostrum; knob: Ridge‐like protrusion located on the dorsal side of the otolith; leafed aragonite: Lamellar projection located on the ventral side of the otolith.

The four‐eyed sleeper ( *Bostrychus sinensis* , Lacepède, 1801) is a fish widely distributed along the Indo‐Pacific Ocean and is also found in the coastal waters of southeastern China (Wu and Zhong 2008). It inhabits the intertidal zone, exhibits a broad salinity tolerance ranging from 0‰ to 35‰, and occurs in coastal, brackish, and estuarine environments (Zhu et al. 2000; Peh et al. 2009). The sister new species *B. donghaiensis* was only recently described from the East China Sea, north of the Taiwan Strait (Zhang et al. 2024). Genetic evidence suggests that sea level fluctuations during the glacial cycles led to isolation and subsequent secondary contact between the two sister species (Qiu et al. 2016; Ding et al. 2019). In their contact zone (Figure 1), despite the continuous northward migration of *B. sinensis* from outside the contact zone, the two species are at an advanced stage of speciation with limited gene flow (Ding et al. 2019; Kulmuni et al. 2020). An extensive histological dataset revealed partially overlapping spawning seasons within the contact zone, suggesting that temporal isolation alone is not a sufficient reproductive barrier between *B. sinensis* and *B. donghaiensis* (Wu et al. 2021). Although the sister species *B. sinensis* and *B. donghaiensis* coexist in a contact zone, the ecological strategies that limit gene flow remain unclear. Our study therefore aims to investigate whether ecological reproductive barriers, mediated by spatial or temporal habitat shifts, have driven the divergence of this *Bostrychus* sister‐species pair.

Otoliths are calcium carbonate structures in the inner ear of fish, growing continuously throughout life and possessing the stability of hard tissues, which enables them to reliably record life history and environmental information (Weatherley and Gill 1987; Belay and Mengist 2025). The morphology of otoliths is widely used for fish classification and population identification, while their chemical composition reflects the environmental conditions experienced by the fish (Ben Labidi et al. 2020; Khedher et al. 2021; Bakkari et al. 2024; Houeto et al. 2024). The microchemical analysis of fish otoliths provides new opportunities to investigate ecological shifts across an individual's life history (Sturrock et al. 2015). Element composition analyses from the core to the edge of an otolith therefore provide insights of the different habitats a fish has experienced throughout its life (Sih et al. 2022). Here, advances in analytical techniques, such as laser ablation inductively coupled plasma mass spectrometry (LA‐ICP‐MS), have significantly advanced the resolution and precision to detect microchemical elemental composition in otoliths (Serre et al. 2018). Indeed, even minor changes in the marine environment can lead to differential accumulation of chemical elements in otoliths, allowing to track habitat shifts and migration patterns of marine fish such as dog snapper ( *Lutjanus jocu* ; Menezes et al. 2021), or *Pacific halibut* ( *Hippoglossus stenolepis* ; Loher et al. 2021). Otolith microchemistry analysis typically focuses on elements such as barium (Ba) and strontium (Sr), whose levels are strongly correlated with the concentrations found in the respective environment (Tabouret et al. 2010). Previous studies have shown that Ba is more sensitive to environmental changes than Sr, and that ratios between Ba and calcium are negatively correlated with salinity, making it useful to distinguish between freshwater and marine populations (Elsdon and Gillanders 2005). Similarly, the ratio between Sr and calcium is positively correlated with salinity and is often used to study fish migration across different salinity gradients (Yang et al. 2011). Combined with other elements that are more influenced by factors such as temperature or physiological conditions, including lithium (Li), magnesium (Mg), manganese (Mn), and copper (Cu), otolith microchemistry has the potential to reveal fine‐scale ecological variation within and among individuals as well as populations (Raby 2023).

Taking advantage of the potential of otoliths for species identification and fine‐scale ecological shifts, the phenotypic differences in otolith shape were first quantified for the two *Bostrychus* species in sympatry, as well as *B. sinensis* from outside the contact zone. In the second step, the concentrations of six microchemical elements were measured along the otoliths to uncover potential habitat transitions across different life history stages and assess whether ecological factors could contribute to reproductive isolation in this system.

## 2 Materials and Methods

### 2.1 Sampling and Otolith Morphological Measurement

This study was conducted in the southeastern coastal region of China, an area characterized by its complex topography, numerous islands, and shallow seas (Wang 1999; Figure 1). Salinity variations along the southeastern coastal region are primarily driven by freshwater inputs from the Yangtze and Pearl Rivers, with fluctuations in river discharge directly affecting nearshore salinity levels, which generally decrease with increasing distance from the shore (Delcroix and Murtugudde 2002; Cai et al. 2004). From October to December 2023, specimens of the genus *Bostrychus* were collected from Zhoushan (30°19′27″ N, 122°09′05″ E), Wenzhou (28°19′30″ N, 121°10′30″ E), Ningde (26°51′20″ N, 120°07′10″ E), Beihai (21°21′43″ N, 109°60′53″ E) and the temperature and salinity near the sampling sites were recorded (Table S1). All specimens were collected using fish cage traps at a depth of less than 10 m and were transported to the laboratory in coolers. After selecting individuals that had reached sexual maturity, a total of 49 fish specimens were obtained, including 31 *B. sinensis* and 18 *B. donghaiensis*. Based on the distribution range and evolutionary history of the sampled species, the specimens were divided into three groups: 18 *B. sinensis* from the contact zone (Csin), 13 *B. sinensis* from outside the contact zone (OUTCsin), and 18 *B. donghaiensis* from the contact zone (Cdon) (Figure 1, Table S1). For each specimen, the standard length (SL) of the fish was measured using a vernier caliper. Sagittal otoliths were carefully extracted, cleaned, and air‐dried at room temperature. Standardized microscopic images of the left and right otoliths were captured using a Leica M165FC microscope, ensuring that all otoliths were consistently oriented with the longest axis parallel to the horizontal baseline.

The landmark‐based software TpsDig 2.16 (Rohlf 2015) was employed to measure seven linear morphological parameters of sagittal otoliths (Table S2): length (L), height (H), length‐to‐height ratio (L/H), the distance from the central concave point of the otolith to the excisural notch (L1), the angle between the antirostrum and rostrum (as defined by Messieh 1972 and Gaemers 1983), as well as the area and perimeter of the otolith. The area of the otolith refers to its two‐dimensional projected surface area.

### 2.2 Linear Morphometric Analysis

A pairwise *t*‐test was used to detect differences between the left and right otoliths. A one‐way analysis of variance (ANOVA) was employed to assess standard length differences between groups. Given that differences in individual size could introduce bias into the analysis of otolith shape variation due to ontogenetic allometry (Simoneau et al. 2000). The effects of standard length on otolith morphological parameters were assessed using analysis of covariance (ANCOVA). Variables that exhibited a significant correlation with standard length but no significant interaction among groups were standardized using the common within‐group slope b (Burke et al. 2008; Agüera and Brophy 2011). Adjusted morphological variables were tested for differences among groups and are presented as means ± standard errors. Variables that showed no significant correlation with standard length were analyzed for group differences using one‐way ANOVA. Subsequently, a principal component (PC) analysis was performed on the morphological variables. All statistical analyses were performed using SPSS version 26.0 (IBM Corp., Armonk, NY, USA), and differences were considered statistically significant at *p* < 0.05.

### 2.3 Geometric Morphometric Analysis

Ten landmarks (LMs) were set for each otolith to capture the endpoints along the contour (LM1–LM9) and a central concave point (LM10; Figure 3A). TpsSmall 1.29 (Rohlf 2015) was used to perform least squares regression analyses on the landmark data, calculating the regression coefficients for the tangent space distance (*y*‐axis) and Procrustes distance (*x*‐axis) of the selected landmarks. TpsRelw 1.49 (Rohlf 2010) was further employed for superimposition, magnification, and distortion of the landmark data to obtain the average otolith shape of the three groups, along with relative warp scores. A thin‐plate spline analysis in TpsRegr 1.37 (Rohlf 2009) was subsequently used to visualize the grid plots of the otoliths for the three groups to compare phenotypic differences. Canonical discriminant analysis and cross‐validated discriminant analysis were finally conducted in SPSS 26.0 using the relative warp scores of the three groups to evaluate classification accuracy.

### 2.4 Otolith Microchemical Analysis

A total of 15 samples were randomly selected, including five *B. donghaiensis* from Zhoushan, five *B. sinensis* from Zhoushan, and five *B. sinensis* from Beihai, to reconstruct microhabitat use during individual ontogeny. The otoliths were polished sequentially with sandpapers of 500, 1200, 2400, and 4000 grit using a figure‐eight motion until the core was exposed. After polishing on a polishing machine at 200 rpm for 2 min, the otolith core and growth rings were examined under a microscope (Leica M205 A, Olympus BX‐51). For specimens with indistinct rings, 5% EDTA was applied for repeated acid etching until the rings became clearly visible (Khumbanyiwa et al. 2018). Along the longest axis of the otolith, opaque and translucent bands were counted from the core to the edge, with one opaque band and one translucent band corresponding to one annual growth increment (Claridge and Potter 1983).

Trace element concentrations in otoliths were measured using laser ablation inductively coupled plasma mass spectrometry (LA‐ICP‐MS) at the Laboratory of Fishery Microchemistry, Freshwater Fisheries Research Center, Chinese Academy of Fishery Sciences (Wuxi, China). The laser ablation system used was the NWR213 (New Wave Research Inc., Fremont, CA, USA), and the ICPMS was the Agilent 7500ce ICPMS (Agilent Technologies, Santa Clara, CA, USA). The ablation was performed by continuously scanning from the core to the edge at a rate of 10 μm/s. National Institute of Standards and Technology (NIST) 612 served as an external standard to adjust for instrument mass bias. The analysis involved a 100‐s background count at the beginning and the end to determine the actual limits of detection (LODs) and relative standard deviations (% RSD) based on replicated measurements of the standard sample to assess the analytical precision for each element (Pan et al. 2024). A total of 19 elements were detected in the otoliths, and after applying a screening process based on the limit of detection (LODs) and ensuring that relative standard deviations (% RSD) were below 10%, six elements were identified as effectively detectable for further analysis: barium (Ba), copper (Cu), lithium (Li), magnesium (Mg), manganese (Mn), and strontium (Sr). The other 13 elements were not further considered (Na, Al, K, Ca, Sc, Ti, V, Cr, Fe Co, Ni, Zn, Cd). Due to the significantly lower concentrations of the measured elements compared to calcium (Ca), all results were expressed as concentration ratios of elements to calcium (mmol/mol) in accordance with international conventions (Longerich et al. 1997).

Variations in six elements from the core to the edge of the otoliths were quantified to explore potential habitat shifts across different life history stages. A principal component (PC) analysis was performed on the microchemical element concentrations of all individuals. Averaged values of each element were plotted as a ratio to Ca at 50 μm intervals along the otoliths for each group separately. The Ba/Ca ratio from the core to the edge of each otolith was plotted, and one representative individual from each group was selected based on the otolith's growth characteristics to illustrate changes in barium across life‐history stages.

## 3 Results

### 3.1 Morphological Characteristics of Sagittal Otoliths of *B. sinensis* and *B. donghaiensis*

**FIGURE 2:**
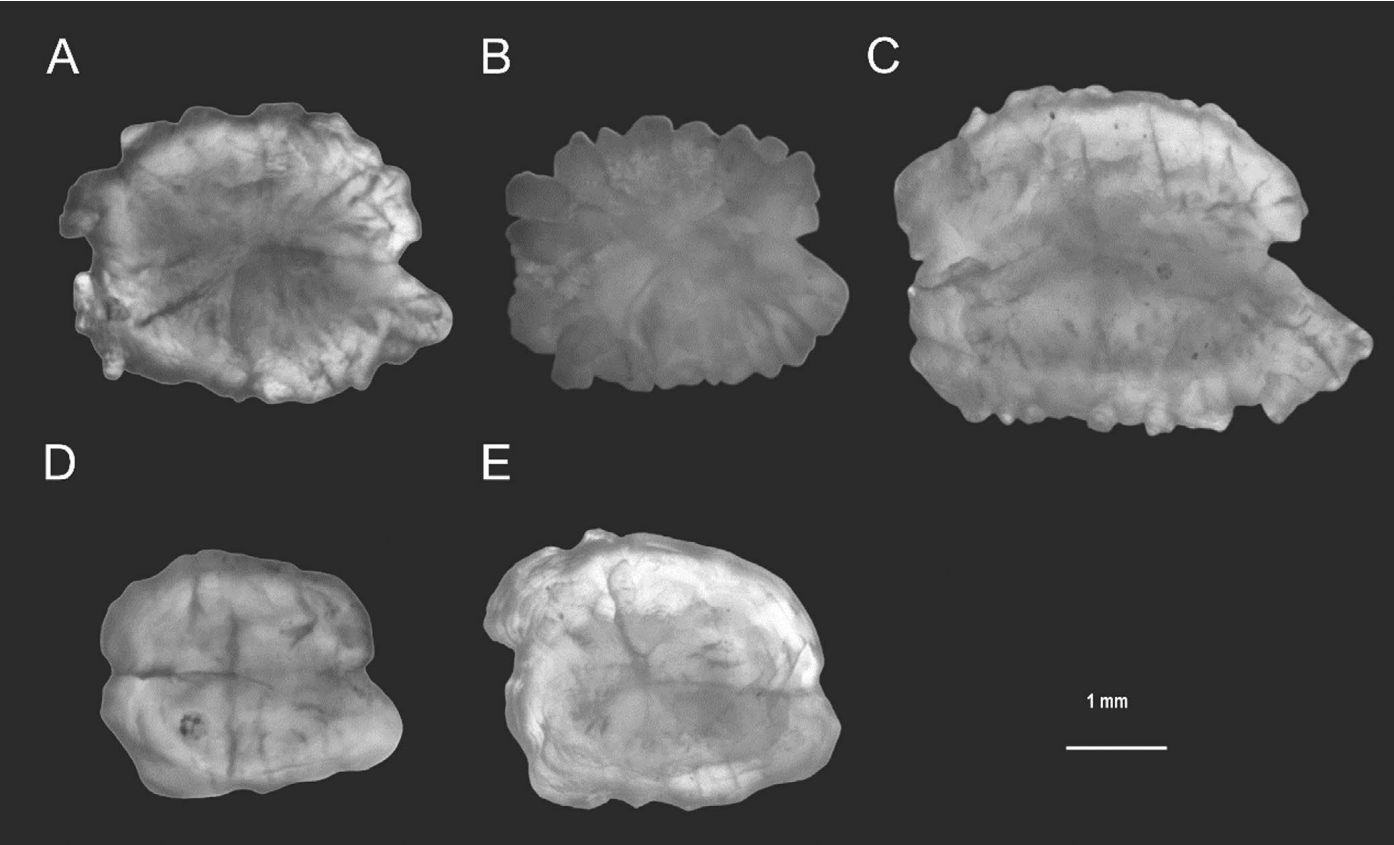
A comparison of the left sagittal otoliths between *B. sinensis* and *B. donghaiensis*. (A–C) *B. sinensis* (standard length, SL: 128.17, 112.01, 151.64 mm), collected from Zhoushan, Wenzhou, and Beihai, respectively. (D, E) *B. donghaiensis* (SL: 113.51 and 123.00 mm), collected from Zhoushan and Ningde.

The samples collected in this study included 31 *B. sinensis* and 18 *B. donghaiensis*. The morphological measurements of the left and right sagittal otoliths showed no significant asymmetry (Tables S2 and S3; *p* > 0.05). Therefore, only the left otolith was used for the subsequent analyses. A qualitative comparison of otolith morphology among samples from different sites revealed morphological differences between *B. sinensis* and *B. donghaiensis* (Figure 2). Otoliths of *B. sinensis* were overall more rectangular, that is, the dorsal length was shorter than the ventral length, forming an excisural notch between the antirostrum and the rostrum. The interior of the otoliths contains numerous striations, and the edges of the otoliths feature many irregular spines. In contrast, the otoliths of *B. donghaiensis* exhibited only prominent horizontal and vertical striations on the interior, with few spines along the edges of the otoliths. The otolith outline was relatively smooth, and both the antirostrum and rostrum were relatively blunt.

### 3.2 Linear Morphometrics

**FIGURE 3:**
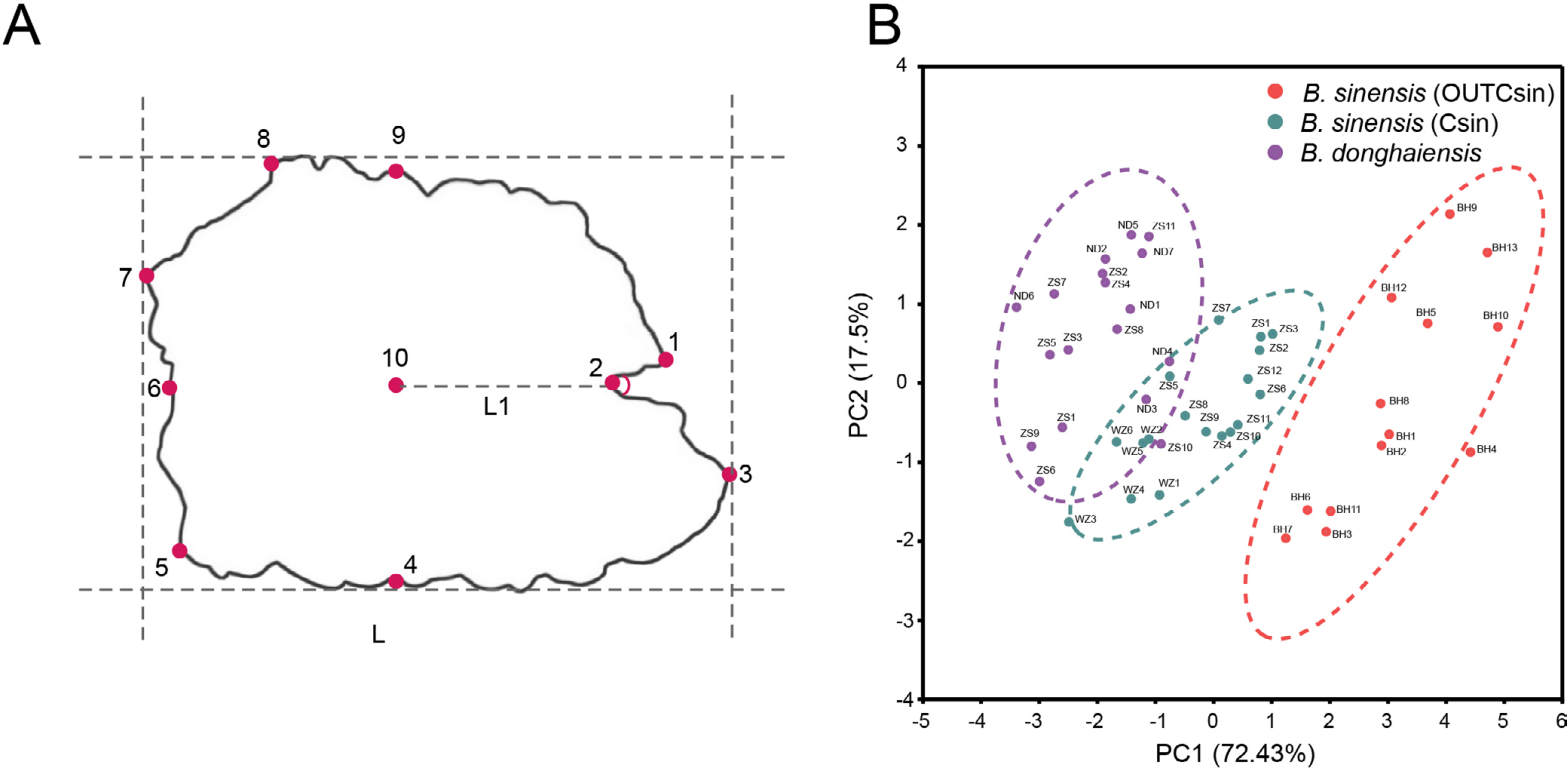
(A) A schematic diagram of morphological measurement parameters and landmarks; (B) Principal component (PC) analysis based on linear morphology.

The collected samples included 18 *B. donghaiensis* from the contact zone (Cdon), 18 *B. sinensis* from within the contact zone (Csin), and 13 *B. sinensis* from outside the contact zone (OUTCsin) (Figure 1, Table S1), consistent with their known distribution and evolutionary background (Zhang et al. 2024). In the comparison of standard length (SL) among the three groups, no significant difference was observed between Csin (118.980 ± 2.663) and Cdon (112.962 ± 2.001), whereas a significant difference was observed between Csin (118.980 ± 2.663) and OUTCsin (147.095 ± 1.584). After correcting for SL using ANCOVA, significant differences were found among the three groups in otolith length (L), height (H), area, and perimeter, whereas no significant difference was observed in L1 (Table S4). The ratio of otolith length to height (L/H) and the angle between the antirostrum and rostrum were not affected by standard length. One‐way ANOVA revealed that L/H differed significantly between Csin (1.278 ± 0.016) and OUTCsin (1.351 ± 0.026), but no significant difference was found between Cdon (1.240 ± 0.014) and Csin (1.278 ± 0.016) (Table S4). For the angle, no significant difference was observed between Csin (84.052° ± 1.846°) and OUTCsin (74.038° ± 4.510°), whereas a significant difference was detected between *B. sinensis* and *B. donghaiensis* (117.897° ± 3.987°). Although a few individuals in all three groups exhibited otolith angles around 90° (Table S2), the overall trend was that the otolith angle in *B. sinensis* was less than 90°, whereas in *B. donghaiensis* it was greater than 90°. A principal component (PC) analysis on the linear traits revealed a clear separation of the three groups along the first PC axis, accounting for 72.43% of the total variance (Figure 3B). PC1 primarily captured variation associated with the length, height, area and perimeter of the otoliths, while PC2 (17.50%) mainly reflects variation related to the angle between the rostrum and antirostrum (Table S5).

### 3.3 Geometric Morphometrics

**FIGURE 4:**
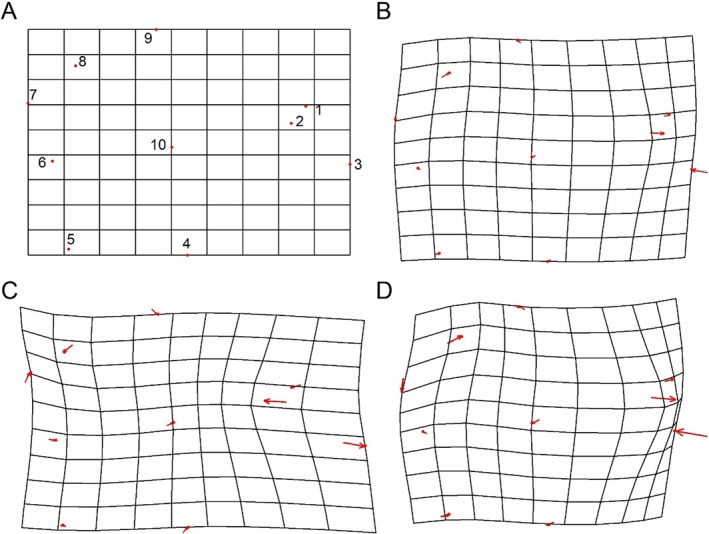
Grid deformations visualizing the variation in otolith shape of *B. sinensis* and *B. donghaiensis* (variation magnified threefold). (A) Average shape of the three groups; (B) *B. sinensis* (Csin); (C) *B. sinensis* (OUTCsin); (D) *B. donghaiensis*.

The study further employed geometric morphometric analysis to differentiate the otolith morphology of the three groups. Ten representative landmarks were digitized for each otolith (Figure 3A). The regression coefficient between the tangent space distance and the Procrustes distance was 0.998, indicating that the selected landmarks were reliable. Based on the relative warp scores, the average shape of the sagittal otoliths was visualized (Figure 4A, Table S6). The relative contributions of each landmark (LM) to the warping process were especially high for LM1 (34.02%) and LM2 (45.37%) (Table S7), which are related to the position of the antirostrum and rostrum. The differences were threefold magnified to obtain the landmark grid plots of sagittal otoliths for the three groups (Figure 4B–D). Here, LM2 of Csin and *B. donghaiensis* showed a shift towards the anterior ventral, with *B. donghaiensis* showing a more pronounced shift, as reflected in the shorter distance between LM2 and LM3 in *B. donghaiensis*. In OUTCsin, LM3 shifted more towards the anterior ventral, whereas LM3 in Csin and *B. donghaiensis* shifted towards the posterior dorsal.

**FIGURE 5:**
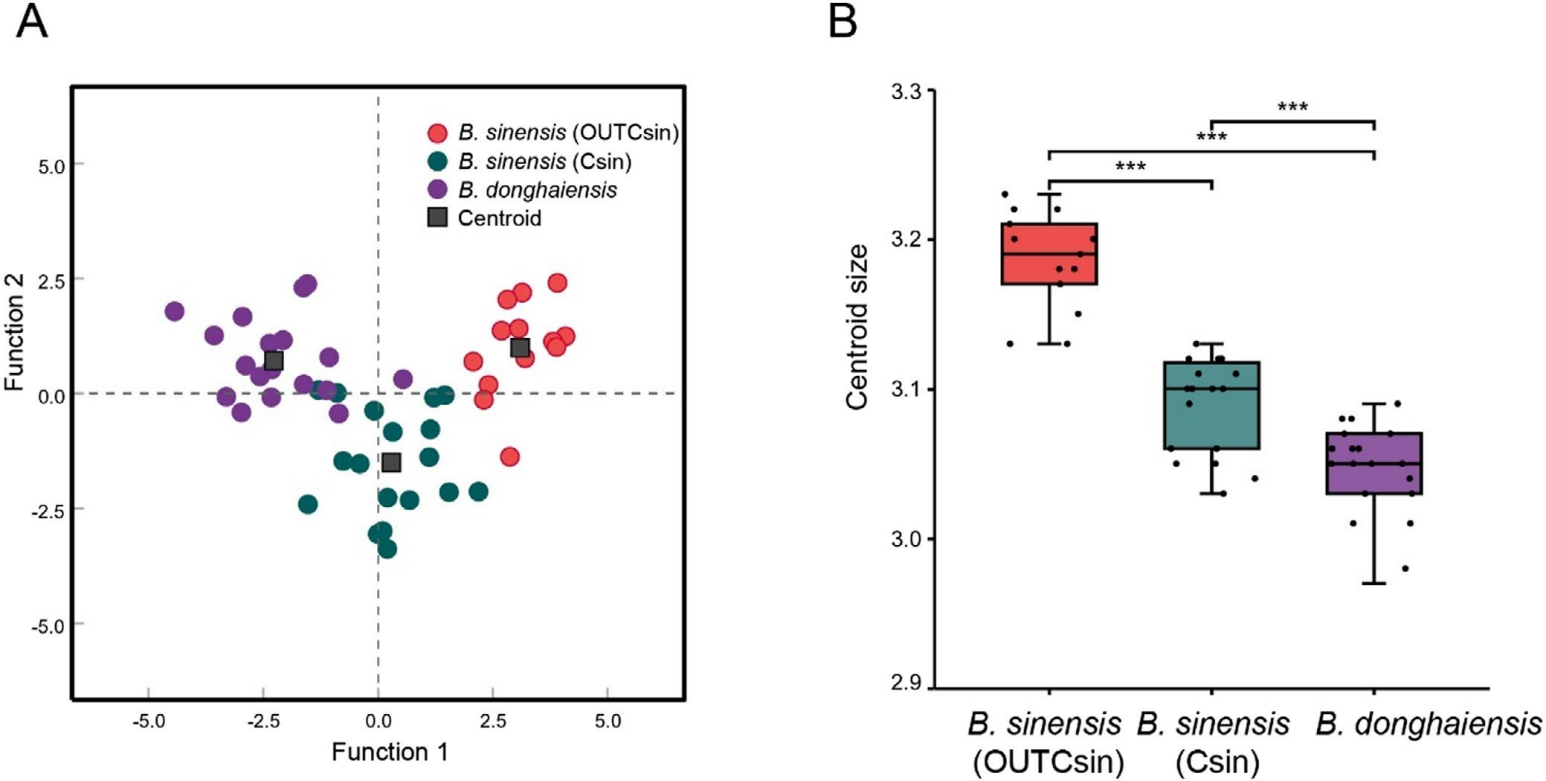
(A) Scatter plot of a canonical discriminant analysis among the three groups. (B) Box plot of centroid size of otolith shapes among the three groups; *** denotes a significance level of *p* < 0.001.

The relative warp scores showed no evidence of multicollinearity, indicating that the selected variables are independent. By performing discriminant function analysis combined with cross‐validation analysis using all relative warp scores, high assignment probabilities for the three groups were found (Table 1). In the discriminant function analysis, the assignment probability for OUTCsin was 100%, while a few individuals were misclassified between Csin and *B. donghaiensis*, with assignment probabilities of 88.9% for both Csin and *B. donghaiensis*. In the cross‐validation results, the assignment probability for OUTCsin was 76.9%, while that for Csin was 61.1%, and *B. donghaiensis* had the highest assignment probability (83.3%; Table 1). In the scatter plot of canonical discriminant function analysis, the three groups were clearly distinguished (Figure 5A). Centroid size differed among the three groups, with OUTCsin and *B. donghaiensis* being the most distinct (Figure 5B).

**TABLE 1:** Summary of a discriminant and cross‐validation analysis of otolith morphology.

Method	Predicted species	Discriminated species			*N*
		*B. sinensis* (OUTCsin)	*B. sinensis* (Csin)	*B. donghaiensis*	
Discriminant	*B. sinensis* (OUTCsin)	13 (100%)	0 (0%)	0 (0%)	13
	*B. sinensis* (Csin)	0	16 (88.9%)	2 (11.1%)	18
	*B. donghaiensis*	0	2 (11.1%)	16 (88.9%)	18
Cross‐Validation	*B. sinensis* (OUTCsin)	10 (76.9%)	3 (23.1%)	0 (0%)	13
	*B. sinensis* (Csin)	3 (16.7%)	11 (61.1%)	4 (22.2%)	18
	*B. donghaiensis*	0 (0%)	3 (16.7%)	15 (83.3%)	13

### 3.4 Reconstructing Habitat Using Otolith Microchemistry

**FIGURE 6:**
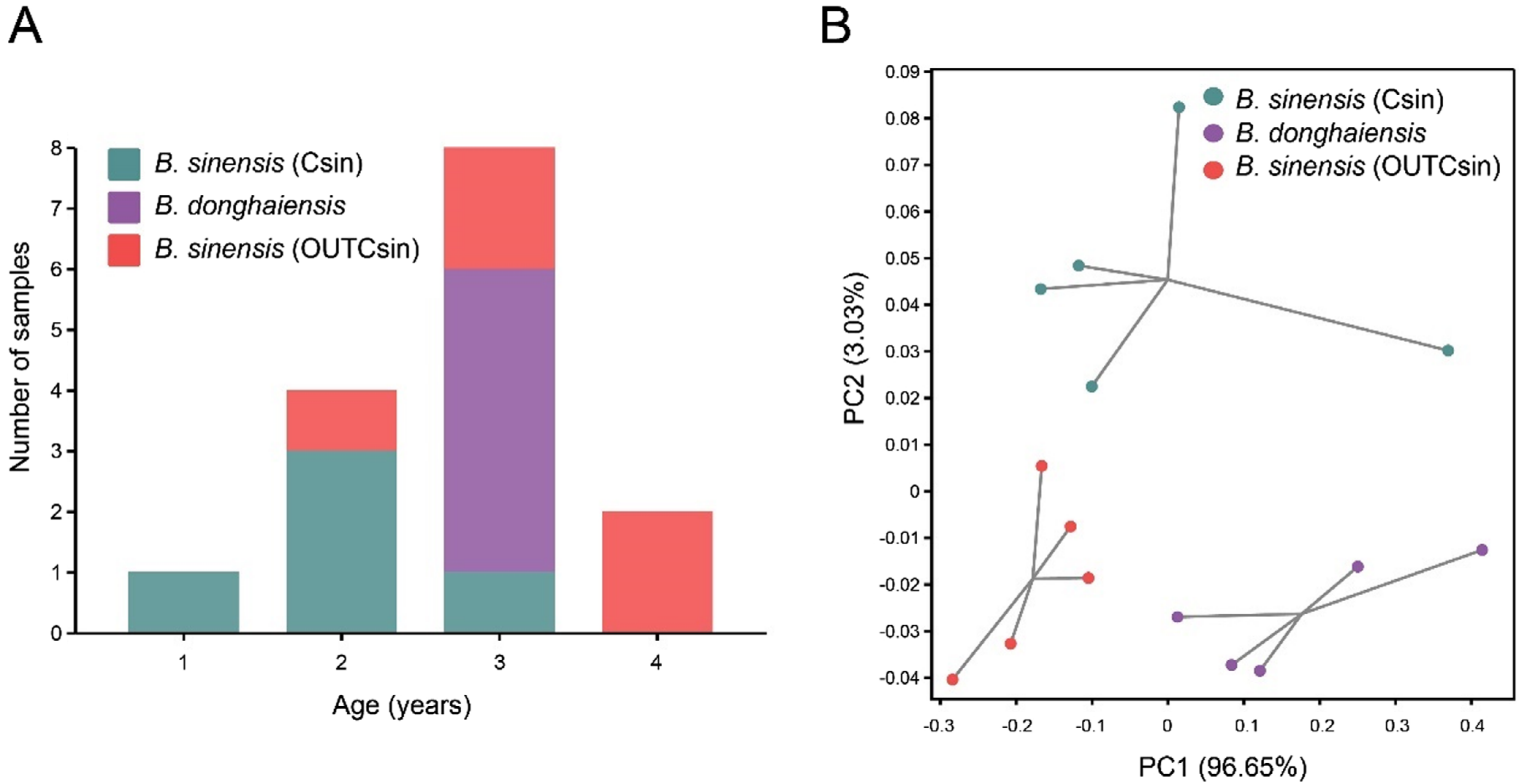
(A) Age composition (in years) of the studied specimens. (B) Principal component (PC) analysis of otolith microchemistry.

At the Zhoushan and Beihai sampling sites, five individuals from each group were randomly selected to conduct otolith microchemical element analysis (Table S8), with the aim of reconstructing the microhabitat conditions during their individual developmental processes. Otolith age readings indicate that the ages of the fish are primarily concentrated at 2 and 3 years, with the mean ages being 2 years for Csin, 3 years for *B. donghaiensis*, and 3.2 years for OUTCsin (Figure 6A). The mean values of six elements detected from the otolith core to the edge were obtained for each group and expressed as the ratio of each element to calcium (X/Ca, mmol/mol). Except for Li and Cu, the mean values of Mg, Mn, Sr, and Ba showed differences among the three groups (Table 2). Principal component (PC) analysis of the six chemical elements in all samples revealed a clear separation among the three groups (Figure 6B). Here, the first principal component (PC1) axis explained 96.65% of the total variation, effectively separating *B. donghaiensis* from OUTCsin. The second PC axis (PC2), accounting for 3.03% of the variation, distinguished Csin from both *B. donghaiensis* and OUTCsin (Figure 6B).

**TABLE 2:** Average elements‐to‐calcium ratios (X/Ca, mmol/mol) for each group (± SD).

Elements	*B. sinensis* (Csin)	*B. donghaiensis*	*B. sinensis* (OUTCsin)
Li/Ca	0.003 ± 0.002	0.003 ± 0.005	0.003 ± 0.002
Mg/Ca	0.153 ± 0.068	0.086 ± 0.044	0.100 ± 0.054
Mn/Ca	0.043 ± 0.073	0.018 ± 0.019	0.003 ± 0.003
Cu/Ca	0.002 ± 0.001	0.002 ± 0.002	0.002 ± 0.001
Sr/Ca	3.275 ± 0.953	3.093 ± 0.875	3.450 ± 0.989
Ba/Ca	0.010 ± 0.006	0.002 ± 0.001	0.002 ± 0.002

**FIGURE 7:**
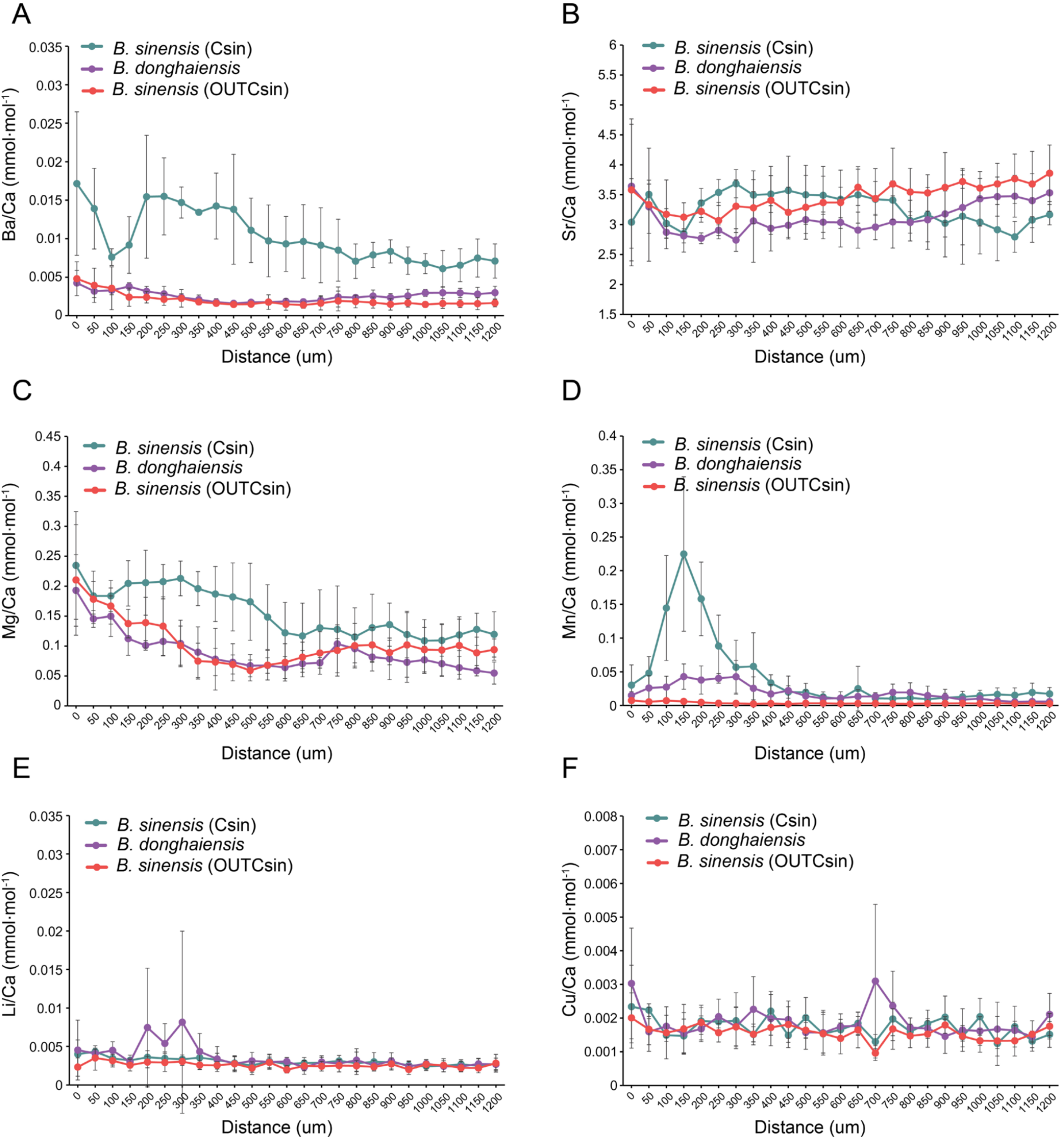
(A–F) Trends in Ba/Ca, Sr/Ca, Mg/Ca, Mn/Ca, Li/Ca, and Cu/Ca ratios from the core to the edge of sagittal otoliths for each group.

The variation in each element during growth was compared, that is, along each otolith from its core to the edge. The profiles were overall consistent with life history difference among the studied groups. Four elements (Ba, Sr, Mg, and Mn) exhibited similar trends in *B. donghaiensis* and OUTCsin (Figure 7A–D), but displayed completely different patterns in Csin. The remaining two elements, Li and Cu (Figure 7E,F) showed similar distribution patterns in Csin and OUTCsin but differed between *B. donghaiensis* and *B. sinensis* .

**FIGURE 8:**
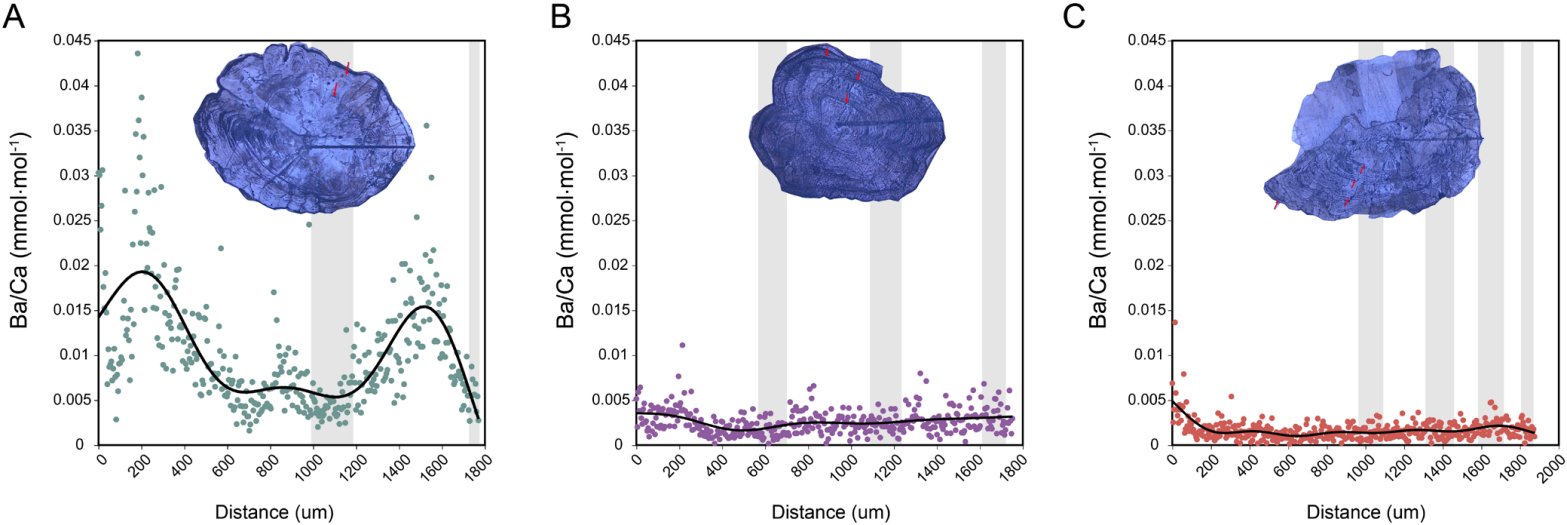
Ba/Ca trajectories of otoliths from three representative individuals. (A) Individual ZS6, Age 2, *B. sinensis* (Csin); (B) Individual ZS2, Age 3, *B. donghaiensis*; (C) Individual BH4, Age 4, *B. sinensis* (OUTCsin). The small red arrows indicate the position of the annular ring in the otolith. The horizontal axis indicates the distance from the core along the otolith. Vertical gray shaded area indicates winter growth, non‐shaded indicates summer growth.

The Ba/Ca ratio in Csin consistently exceeded 0.008 mmol/mol, remaining higher than in the other two groups while showing a fluctuating pattern (Figure 7A). In contrast, *B. donghaiensis* and OUTCsin showed Ba/Ca ratios of less than 0.005 mmol/mol, which remained relatively stable throughout growth (Figure 7A). The variation in Ba concentrations from the core to the edge of each individual was examined, revealing that Csin individuals exhibited periodic peaks in Ba levels, with the number of peaks corresponding to the individual's age. In contrast, no obvious periodic fluctuations in Ba concentrations were observed in the OUTCsin and *B. donghaiensis* (Figure S1). Focusing on representative individuals, an analysis of the Ba/Ca ratios was conducted at a finer resolution (Figure 8). In Csin, the Ba/Ca ratios (0.004–0.02) exhibited two pronounced peaks that corresponded to the spring and summer phases of its annual life cycle (Figure 8A). In contrast, *B. donghaiensis* from the contact zone exhibited consistently stable Ba/Ca ratios (around 0.0025) throughout its growth process (Figure 8B). In OUTCsin, Ba/Ca ratios (around 0.0015) also remained relatively constant throughout its growth process (Figure 8C). In addition, the Sr/Ca ratio in Csin was observed to exceed those of the other two groups at around 200 μm, followed by a decline near 700 μm (Figure 7B). In contrast, the Sr/Ca ratios in the otoliths of *B. donghaiensis* and OUTCsin remained relatively stable throughout the entire growth process.

For Mg and Mn, the Mg/Ca ratio in Csin was observed to initially rise slowly, then decline before stabilizing, while in *B. donghaiensis* and OUTCsin, a rapid decline was observed followed by stabilization (Figure 7C). A significant peak in Mn concentration was observed near the core of the otolith in Csin at 150 μm, after which the concentration decreased to levels comparable to those of the other two groups (Figure 7D), while Mn concentration in the other two groups remained consistently stable.

The Li/Ca and Cu/Ca ratios differed only between *B. donghaiensis* and *B. sinensis* , while exhibiting identical trends in Csin and OUTCsin. In the range of 150–350 μm, Li/Ca levels in *B. donghaiensis* fluctuated, while those in *B. sinensis* remained relatively constant (Figure 7E). For Cu/Ca, *B. donghaiensis* exhibited a prominent peak at approximately 700 μm from the otolith core, whereas *B. sinensis* showed a trough at the same position (Figure 7F).

## 4 Discussion

Overall, the otolith outlines showed obvious differences among the three groups. In this study, differences in the otolith L/H ratio were observed between the Csin and OUTCsin groups of *Bostrychus sinensis* , whereas differences in the angle between the antirostrum and rostrum were observed between *B. sinensis* and *B. donghaiensis*. Otolith morphology can be used not only to characterize intraspecific variation within *B. sinensis* , but also to provide supplementary interspecific traits that may facilitate species discrimination between *B. sinensis* and *B. donghaiensis* (Figure 2). At an equivalent standard length, *B. donghaiensis* individuals were consistently older than *B. sinensis* from the contact zone, indicating a faster growth rate in *B. sinensis* , consistent with previous observations that *B. donghaiensis* tends to be smaller (Zhang et al. 2024). The slower growth of *B. donghaiensis* during the juvenile stage was also evident in the shorter distance from the otolith core to the first annulus, which formed at approximately 600 μm (Figure 8B), whereas in *B. sinensis* the first annulus appeared at around 1000 μm (Figure 8A,C). According to Zhang (2002), *Bostrychus sinensis* populations consisted of only four age classes, with the period of rapid growth occurring before sexual maturity. Considering that age may potentially influence comparisons of otolith morphology, otolith shape was size‐corrected using fish standard length in this study. Although otolith age estimation indicated that the mean age of Csin (2 years) was lower than that of *B. donghaiensis* (3 years), interspecific differences in otolith morphology appeared to be greater than the ontogenetic variations associated with age. Future research with a larger sample size may help clarify the morphological differences among different age stages within the genus *Bostrychus*.

While genetic and environmental factors may provide plausible explanations for the observed morphological differences in otoliths (Neves et al. 2025), further otolith microchemical analysis could help elucidate the habitat characteristics during the life history processes of diverged species or lineages. The preliminary PCA clustering results based on six otolith microchemical elements revealed distinct microchemical signatures among the three *Bostrychus* species groups (Figure 6B), which are consistent with the intra‐ and interspecific genetic differentiation observed in previous studies (Qiu et al. 2016). Trace elements in the aquatic environment may interact with each other, and the concentrations of different elements reflect specific physicochemical properties of the water. For example, Sr facilitates Ba uptake (De Vries et al. 2005), and the deposition of both elements in otoliths plays a key role in tracking transitions between freshwater and saltwater in fish (Elsdon and Gillanders 2005; Walther and Thorrold 2006). The Ba concentrations in otoliths exhibited substantial variation along the estuary‐marine gradient and showed a negative correlation with salinity, making them a key indicator for detecting habitat shifts. Xu et al. (2023) inferred habitat use of large yellow croaker ( *Larimichthys crocea* ) in the East China Sea using Ba/Ca ratios, with values below 0.0042 mmol/mol indicating marine habitats, values above 0.0081 mmol/mol indicating estuarine habitats, and values between 0.0042 and 0.0081 mmol/mol indicating estuary‐marine mixed habitats. In this study, Ba/Ca ratios (< 0.005) were consistently low and stable in OUTCsin and *B. donghaiensis*, whereas those in Csin exceeded 0.008 mmol/mol and fluctuated, showing a pattern inconsistent with species classification (Figure 7A). Notably, periodic Ba/Ca peaks were consistently observed in each individual of the Csin group, and the number of peaks was in agreement with their estimated ages (Figure S1). To further investigate the changes in Ba/Ca concentrations in representative individuals, a two‐year‐old Csin exhibited two distinct Ba/Ca peaks throughout its life history, with the highest peak reaching 0.02 and baseline values around 0.004 (Figure 8A), indicating a shift in habitat from high‐salinity waters to low‐salinity waters during its life history. The Ba/Ca peaks occurred during the spring–summer spawning season, suggesting seasonal reproductive migration to low‐salinity estuarine habitats. In contrast, consistently low Ba/Ca ratios observed in *B. donghaiensis* and OUTCsin (< 0.0042 mmol/mol; Figure 8B,C) throughout their life history suggest that these individuals resided in high‐salinity marine environments without undergoing migration. The habitat transitions of Csin were further supported by changes in Sr concentrations, which are positively correlated with salinity. In Csin, Sr/Ca ratios were initially very low near the otolith core and subsequently exhibited an increase followed by a decrease, consistent with the hypothesis that larvae hatch in freshwater, migrate to saltwater environments for growth, and return to freshwater for spawning and mating. However, the Sr/Ca ratios in the otoliths of *B. donghaiensis* and OUTCsin remained relatively stable throughout growth, indicating the absence of habitat migration behavior (Figure 7B). Other microchemical elements also support this inference, as the observed shifts in Mg/Ca and Mn/Ca ratios in Csin between early and later life stages are consistent with habitat transitions (Figure 7C,D). Additionally, the differences in Mg/Ca ratios between Csin and OUTCsin may suggest variations in temperature, potentially related to latitude (Miller 2011).

If closely related species that are ecologically similar come into secondary contact, then one species is likely to outcompete the other in the long term (Munday 2004). Alternatively, one of the species may undergo ecological character displacement to limit interspecific competition (Pfennig and Pfennig 2009). Utilizing an otolith microchemical analysis to trace the life history processes of *B. sinensis* and *B. donghaiensis* within the contact zone, distinct habitat migration patterns were revealed during the reproductive phase of these two sister species. This is the first demonstration of how ecological isolation contributes to reproductive isolation between *B. sinensis* and *B. donghaiensis* in the contact zone. Notably, intraspecific differences were found in *B. sinensis* , with individuals of the ancestral region south of the Taiwan Strait exhibiting a reproductive strategy similar to that of *B. donghaiensis*. In contrast, *B. sinensis* in the contact zone displayed a reproductive migration pattern from high to low salinity waters. This suggests that the change in reproductive strategy of *B. sinensis* occurred following secondary contact. This is consistent with ecological character displacement, potentially to reduce interspecific competition and potential hybridization. Furthermore, the low‐salinity estuarine environments in the contact zone provide suitable breeding habitats for *B. sinensis* . The spatial shift during the reproductive phase not only allows Csin to exploit a greater range of ecological resources throughout its life history but also helps to physically avoid gene flow with its sister species. Future research could further investigate the breeding habitats of different groups of *B. sinensis* and *B. donghaiensis* by conducting sampling surveys in aquatic environments with varying salinity levels.

Taken together, both interspecific and intraspecific differences in otolith morphology and microchemical composition were observed between the two sister species, *B. sinensis* and *B. donghaiensis*. The different otolith‐based approaches provided novel insights into how these species utilize their habitats and avoid niche overlap in their zone of secondary contact, that is, likely through ecological character displacement. These findings shed light on how microhabitat changes in the marine environment promote species coexistence, providing an analytical framework that is broadly applicable to other fish species.

## Author Contributions

**Rongrong Zhang:** data curation (lead), formal analysis (equal), methodology (equal). **Kay Lucek:** conceptualization (equal), writing – original draft (equal). **Lisheng Wu:** formal analysis (equal), methodology (equal). **Shufang Liu:** resources (equal), supervision (equal). **Wenwen Zhang:** data curation (equal). **Hongjun Guo:** data curation (equal). **Shaoxiong Ding:** supervision (lead).

## Funding

This work was supported by the National Natural Science Foundation of China (Grant No. 41776143), the China Agriculture Research System of MOF and MARA (CARS‐47). K.L. was supported by the Swiss National Science Foundation (Grant No. 202869).

## Ethics Statement

This work was approved by the Institutional Animal Care and Use Committee (IACUC) of Xiamen University. All experiments were performed according to the Guidelines for the Care and Use of Laboratory Animals in China.

## Conflicts of Interest

The authors declare no conflicts of interest.

## Supporting information


**Figure S1:** Variation in Ba concentration from the otolith core to the edge in three groups. A: *B. sinensis* from the contact zone (Csin, ZS population); B: *B. donghaiensis* (ZS population); C: *B. sinensis* from outside the contact zone (OUTCsin, BH population).

**Table S1:** Sample information and environmental characteristics of collection sites in this study.
**Table S2:** Sample morphological parameter information of the *Bostrychus* in this study.
**Table S3:** Paired Sample T‐Test of Left and Right Sagittal Otolith Morphological Parameters.
**Table S4:** Comparison of body length and otolith morphometric parameters among the three groups.
**Table S5:** Component matrix of otolith principal component analysis.
**Table S6:** Relative distortion scores based on landmark methods.
**Table S7:** Contribution rates of different landmark.
**Table S8:** Otolith microchemical element data.

## Data Availability

The authors confirm that the data supporting the findings of this study are available in the Supporting Informations.
