# Supplementary material for: Microchemical Analyses of Otoliths Reveal Habitat Differentiation Between a Sympatric Species Pair in the Coastal Waters of China
Source: Ecol Evol. 2026 Apr 1;16(4):e73362. doi: 10.1002/ece3.73362 (PMC13045312; doi:10.1002/ece3.73362)
Supplement: Supplementary file 1 — Figure S1: Variation in Ba concentration from the otolith core to the edge in three groups. A: B. sinensis from the contact zone (Csin, ZS population); B: B. donghaiensis (ZS population); C: B. sinensis from outside the contact zone (OUTCsin, BH population). [file ECE3-16-e73362-s002.docx]

Fig. S1. Variation in Ba concentration from the otolith core to the edge in three groups. A: *B. sinensis* from the contact zone (Csin, ZS population); B: *B. donghaiensis* (ZS population); C: *B. sinensis* from outside the contact zone (OUTCsin, BH population).
